# Neural Activity During Audiovisual Speech Processing: Protocol For a Functional Neuroimaging Study

**DOI:** 10.2196/38407

**Published:** 2022-06-21

**Authors:** András Bálint, Wilhelm Wimmer, Marco Caversaccio, Stefan Weder

**Affiliations:** 1 Department of Otorhinolaryngology, Head and Neck Surgery Inselspital, Bern University Hospital, University of Bern Bern Switzerland; 2 Hearing Research Laboratory ARTORG Center for Biomedical Engineering Research, University of Bern Bern Switzerland

**Keywords:** hearing loss, brain plasticity, functional near-infrared spectroscopy (fNIRS), cochlear implant, neuroimaging, speech understanding, comprehension, speech, brain activation, brain activity, hearing impairment, cortical activation, neural, brain, protocol, spectroscopy, cochlear, hearing

## Abstract

**Background:**

Functional near-infrared spectroscopy (fNIRS) studies have demonstrated associations between hearing outcomes after cochlear implantation and plastic brain changes. However, inconsistent results make it difficult to draw conclusions. A major problem is that many variables need to be controlled. To gain further understanding, a careful preparation and planning of such a functional neuroimaging task is key.

**Objective:**

Using fNIRS, our main objective is to develop a well-controlled audiovisual speech comprehension task to study brain activation in individuals with normal hearing and hearing impairment (including cochlear implant users). The task should be deductible from clinically established tests, induce maximal cortical activation, use optimal coverage of relevant brain regions, and be reproducible by other research groups.

**Methods:**

The protocol will consist of a 5-minute resting state and 2 stimulation periods that are 12 minutes each. During the stimulation periods, 13-second video recordings of the clinically established Oldenburg Sentence Test (OLSA) will be presented. Stimuli will be presented in 4 different modalities: (1) speech in quiet, (2) speech in noise, (3) visual only (ie, lipreading), and (4) audiovisual speech. Each stimulus type will be repeated 10 times in a counterbalanced block design. Interactive question windows will monitor speech comprehension during the task. After the measurement, we will perform a 3D scan to digitize optode positions and verify the covered anatomical locations.

**Results:**

This paper reports the study protocol. Enrollment for the study started in August 2021. We expect to publish our first results by the end of 2022.

**Conclusions:**

The proposed audiovisual speech comprehension task will help elucidate neural correlates to speech understanding. The comprehensive study will have the potential to provide additional information beyond the conventional clinical standards about the underlying plastic brain changes of a hearing-impaired person. It will facilitate more precise indication criteria for cochlear implantation and better planning of rehabilitation.

**International Registered Report Identifier (IRRID):**

DERR1-10.2196/38407

## Introduction

### Background

Disabling hearing loss is a major communication and health problem that affects over 6% of the overall population and over 50% of adults above the age of 65. For adults, deafness leads to social isolation, unemployment, and reliance on social services. This problem will increase with demographic change. It is estimated that by 2050, 10% of the global population will be living with disabling hearing loss [1]. In patients with severe to profound hearing loss, a cochlear implant (CI) offers an effective treatment [2]. A CI is a neuroprosthetic device that electrically stimulates the auditory nerve in response to acoustic stimulation. CIs enable deaf patients to regain their speech understanding [3,4], improve sound localization [5], and increase their quality of life [6]. However, hearing outcomes after implantation surgery vary widely in both prelingually and postlingually deafened patients. About 20%-30% of postlingually deafened patients who receive a CI do not gain the expected benefit from the implant. Nowadays, over 75% of the variance in CI outcomes remains unclear [7-9]. Consequently, it is not possible to predict preoperatively how well a CI candidate will perform with the implant. Therefore, there is an urgent need to better understand this variability and find ways to improve outcomes for people with poor language comprehension.

In the absence of auditory input, the sensory deprivation induces reallocation of cortical areas (so-called brain plasticity). This leads to functional reorganization within the auditory and auditory-related brain cortex, with new functions being assigned to these brain regions [10]. As an example, the visual takeover (also referred as cross-modal reorganization) in the impaired auditory brain areas has been demonstrated. It means that visual information, for instance, during a lipreading task, can be processed partially in former auditory associated brain areas [11-14]. A CI can counteract these hearing loss–induced plastic changes, and the success of the rehabilitation depends on them. It has been shown that different hearing outcomes after implantation correlate with these reorganization processes [3,15-17].

We use functional imaging to study these described plastic brain changes. However, in CI recipients, there are important considerations to make. Despite the efforts of CI manufacturers to allow structural magnetic resonance imaging (MRI) with a surgically implanted device, the technique has limitations. The outer speech processor cannot be worn during MRI scanning and thus cannot be used to assess evoked auditory responses associated with functional MRI. Furthermore, the implanted magnet and electrode array of the CI cause imaging artifacts in MRI and stimulation artifacts in electroencephalography (EEG) [18-20].

Functional near-infrared spectroscopy (fNIRS), on the other hand, is ideal for this patient population [21]. The technique uses near-infrared light to measure the blood oxygen saturation of the cerebral cortex. This allows indirect conclusions to be drawn about neuronal activation. Other advantages of fNIRS are that the measurements are not affected by electrical pulses, do not interfere with the CI, are quiet (which is important in auditory tasks), are noninvasive, suitable for all ages, and enable the evaluation of responses to spoken words and whole sentences.

Previous fNIRS studies with implanted adults showed evidence of cortical reorganization. However, when comparing study findings, there are contradictory results. For example, some studies suggest that strong activation of the auditory cortex during lipreading tasks is a negative predictor of speech understanding with the implant [22,23]. Other publications describe an opposite effect or no effect [24,25].

According to a recent review on fNIRS measurements in CI patients, at the current stage, it is difficult to draw a general conclusion about the potential positive or negative effects of cortical reorganization. Instead, methodological aspects must first be clarified [26]. The effect of cross-modal plasticity may be more complex than suggested in previous studies. One problem with measuring functional brain activation is that many variables need to be controlled. For example, it makes a remarkable difference how patients are selected (pre- or postlingually deafened) [24], whether a study participant is actively engaged in the experiment (otherwise mind wandering might occur) [27], how the stimuli are presented, and whether the task performance is monitored [28]. Poorly controlled variables during an fNIRS experiment can lead to misinterpretations and mistakes in data analysis.

The aim of our study protocol is to develop a well-controlled and reproducible fNIRS task to evaluate brain activation in response to speech comprehension in individuals with normal hearing, those with hearing impairments, and CI users. Our hypothesis is that through such a task, we can identify cortical networks that are clearly correlated to hearing performance with the implant. Identified brain activation patterns may later be used preoperatively as biomarkers of speech understanding with the implant.

### Objectives

Using fNIRS, our main objective is to develop an audiovisual speech comprehension task to measure functional brain activity regarding speech understanding. The task should comply with the following criteria: it should (1) be deducible from clinically established hearing tests; (2) induce maximal cortical activation (and thus allow reproducible recognition of activation patterns); (3) align with the international 10-10 system of electrode placement, using optimally spaced optode positions with maximal coverage over the responsible brain regions. Short separation channels should allow noise reduction; (4) be time-efficient (to avoid fatigue due to experiment duration; (5) be suitable for normal hearing, hearing impaired, and cochlear implant users; and (6) be reproducible by other research groups.

We will correlate the fNIRS recordings with (1) data from patients’ history, (2) clinically validated questionnaires, and (3) performance during the fNIRS measurements (eg, speech comprehension during the fNIRS task).

## Methods

### Study Design

This research project is a prospective single-center study and will be conducted at the Department of Otolaryngology, Head and Neck Surgery at the Bern University Hospital, Inselspital, Bern, Switzerland.

### Ethics Approval

The protocol was designed in accordance with the ethical principles of the Declaration of Helsinki. The study setup was approved by the local ethical committee (reference number 2020-02978) and fulfils all the patient data regulations of Switzerland.

#### Participants and Eligibility Criteria

All study participants must (1) be at least 18 years old, (2) be native German speakers, and (3) have preferably light and thin hair [29,30]. Participants with a severe cardiac, psychiatric, or neurological disease (eg, epilepsy) or brain injury will be excluded from the study (refer to Multimedia Appendix 1 for details). CI users must be bilaterally and postlingually deafened, with an unaided pure-tone average (PTA) hearing threshold exceeding a hearing level (HL) of 80 dB.

The ear through which the acoustic stimulation will be presented needs to be implanted for at least 1 year. This will ensure that hearing rehabilitation after implantation is completed.

Participants will be allocated to one of 3 groups: (1) normal hearing “control” cohort, (2) CI users with good speech understanding (“overperformer”), or (3) CI users with poor speech understanding (“underperformer”). CI users with moderate speech perception (ie, between 40% and 70% aided monosyllabic word recognition score) will not be recruited because we want to investigate the functional mechanisms specifically for good and poor outcomes. Table 1 provides an overview of the categorization criteria for each subgroup.

**Table 1:** Overview of categorization according to participants’ hearing performance^a^.

Criterion	Normal hearing	CI^b^ “overperformer”	CI “underperformer”
Unaided PTA^c^ hearing threshold	≤20 dB HL^d^	≥80 dB HL	≥80 dB HL
Word recognition score	100%	≥70%	≤40%

^a^Word recognition score will be measured using Freiburg monosyllabic test lists at a 65 dB sound pressure level.

^b^CI: cochlear implant.

^c^PTA: pure-tone average.

^d^HL: hearing level.

#### Sample Size

Pilot measurements were performed on 10 participants to estimate an appropriate sample size. We compared the median relative change in the concentration of oxygenated hemoglobin in the auditory cortex. After acoustic stimulation (speech in quiet), an increase of 1.315 (SD 1.275) µMolar*cm was measured, while during the resting state, the value fluctuated close to 0. A power analysis to test a 2-sided hypothesis at 95% significance and 80% power showed that we need at least 15 participants with normal hearing to detect auditory activations. In addition, we considered previous findings from auditory fNIRS studies [26,28,31,32]. We compared the size of their study cohorts, the fNIRS systems used, the optode arrangements used, and the reliability of their results. To allow for a possibly larger variation, we propose including 60 individuals in this study (20 listeners with normal hearing, 20 CI overperformers, and 20 CI underperformers).

#### Recruitment

Recruitment will be done through the CI center of our department. Potential study candidates will be screened based on their medical records and will be subsequently informed verbally or in writing about the study procedure. Candidates who are willing to participate and able to complete all tests required for the study will be asked to sign an informed consent form.

### Study Procedure

Table 2 shows the time schedule for participants. The enrollment and the data collection sessions are described in more detail in the subsequent subsections.

**Table 2:** Overview of the study procedure.

Item	Enrollment session	Data collection session
Information sheet	√	
Medical history	√	
Questionnaires	√	
Hearing tests	√	
fNIRS^a^ recording		√
Behavioral assessment		√
Optode position registration		√
Total duration	30 min	90-120 min

^a^fNIRS: functional near-infrared spectroscopy.

#### Enrollment Session

Potential study candidates will be invited to an enrollment session. First, we will hand out the information sheet and answer any questions the candidates may have. To assess full eligibility, the candidates will have to fill in questionnaires and perform additional hearing tests before data collection. Bilateral CI users will be asked to turn off and remove the audio processor of the worse hearing ear to limit acoustic stimulation exclusively to the better ear. The worse hearing ear will be covered using an ear plug. The full enrollment session will take a maximum of 30 minutes.

##### Questionnaires

Questions on medical history will target the candidates’ handedness (Edinburgh Handedness Inventory) and the presence of diseases, which are among the exclusion criteria [33-35]. Additional questions on health status will inquire about the presence of influences that could alter the brain activity of interest, such as the use of stimulants [36]. CI users will receive questions about the duration of their hearing loss, and if they have tinnitus, about the objectivity and laterality of their tinnitus [37]. The Hearing Ability Questionnaires will investigate lipreading experience and hearing-associated factors, including the Speech, Spatial, and Qualities (SSQ-12) questions [38]. The question sheet should cover the subjective assessment of hearing ability in the last 6 months.

##### Hearing Tests

The audiometric measurements and the fNIRS recordings will take place in an acoustic chamber (6 m × 4 m × 2 m) with a separate ventilation system and electromagnetic shielding. The broadband reverberation time is ~200 ms.

In normal hearing participants, we will assess pure tone air-conduction hearing thresholds with a clinical audiometer (GSI 61, Grason-Stadler). The findings must confirm that participants have no hidden or undetected hearing loss (Table 1). For CI users, audiograms are available from clinical routine measurements.

In all participants, we will measure the word recognition score for Freiburg monosyllabic word lists at a sound pressure level (SPL) of 65 dB [39]. Additionally, we will perform the widely used Oldenburg Sentence Test (OLSA) [40-42]. The sentences will be played with 65 dB SPL background noise, using an adaptive version of the female OLSA test [43-45]. The OLSA sentences will also be used as a stimulus during the fNIRS measurement. Speech material will be presented from a loudspeaker (Control 1 Pro) placed in front of the participants at a distance of 1 m.

#### Data Collection Session

##### Experimental Setup

**Figure 1:**
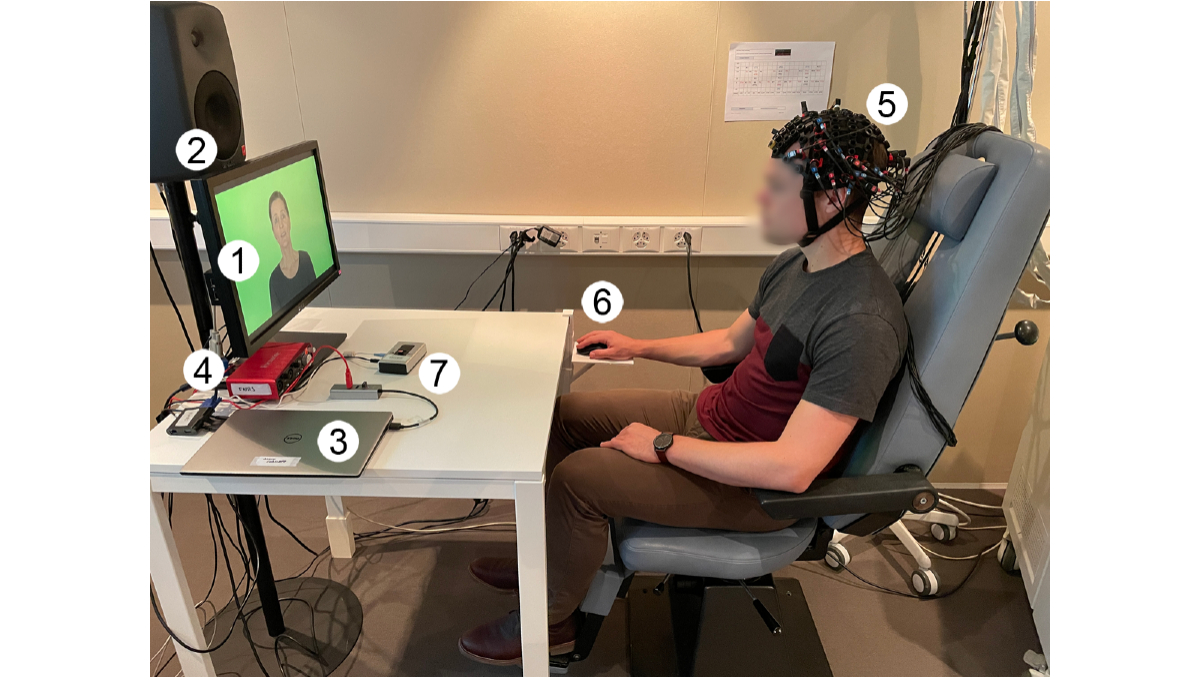
Experimental setup during functional near-infrared spectroscopy (fNIRS) recording. The participant will receive the stimulation via the computer screen (1) and the loudspeaker (2). The loudspeaker will be connected to the control laptop (3) via an external soundcard (4). The fNIRS cap (5) will be fitted on the participant's head, and the subject will interact using a response mouse (6). The alarm button (7) will be positioned in front of the subject.

During fNIRS recording, each study participant will sit in a comfortable chair with an armrest, headrest, and lumbar support (Figure 1). A desk will be placed in front of the participant with the electrical equipment. Visual stimuli will be presented through a computer screen (P2210, Dell) placed on the table at a distance of 120 cm in front of the participant. The acoustic stimuli will be played through a loudspeaker (8040B, Genelec) placed above the monitor at a distance of 130 cm from the ears. The loudspeaker will receive input from an external ASIO sound card (Scarlett 2i2, FocusRite) connected to the control laptop (XPS 13, Dell) via USB. The system will be calibrated to 65 dB SPL with the OLSA calibration noise and an acoustic analyzer (XL2, NTi Audio).

The stimulation protocol will be controlled by a custom-written script (Python 3.8.8) using Tkinter and python-vlc libraries. The script will send triggers via the serial interface to a trigger-box (MMBT-S Interface Box, NEUROSPEC AG), which converts the signals to transistor-transistor Logic (TTL) levels. The TTL-encoded signals will then be received by the fNIRS machine (FOIRE-3000, Shimadzu).

Participants will interact with the control laptop using the buttons of a mouse (WM527, Dell). The pointing function of the mouse will be disabled to ensure that participants control the experiment only by clicking and rolling. During the fNIRS measurement, the participants will be able to press an alarm button (Switchbox, Delock) positioned in a reachable distance on the table.

##### Optode Placement

We will select the regions of interest (ROIs) for the placement of the optodes considering previous studies. We expect responses related to audiovisual speech comprehension in the auditory and visual cortex, more specifically in the following ROIs: superior temporal gyrus (STG), primary visual cortex (V1), and visual association cortex (V2) [28,31,46-50]. Additionally, during similar conditions, the left inferior frontal gyrus (LIFG) has been associated with effortful listening [27,51], and elevated cortical responses have been reported in the middle temporal gyrus (MTG) and middle frontal gyrus (MFG) [52]. Based on the defined ROIs, we will determine the optimal selection of EEG coordinates using the fNIRS optode's location decider (fOLD) toolbox [53]. We will further consider the position of the audio processor and receiver coil in CI participants to avoid interference with optodes.

**Figure 2:**
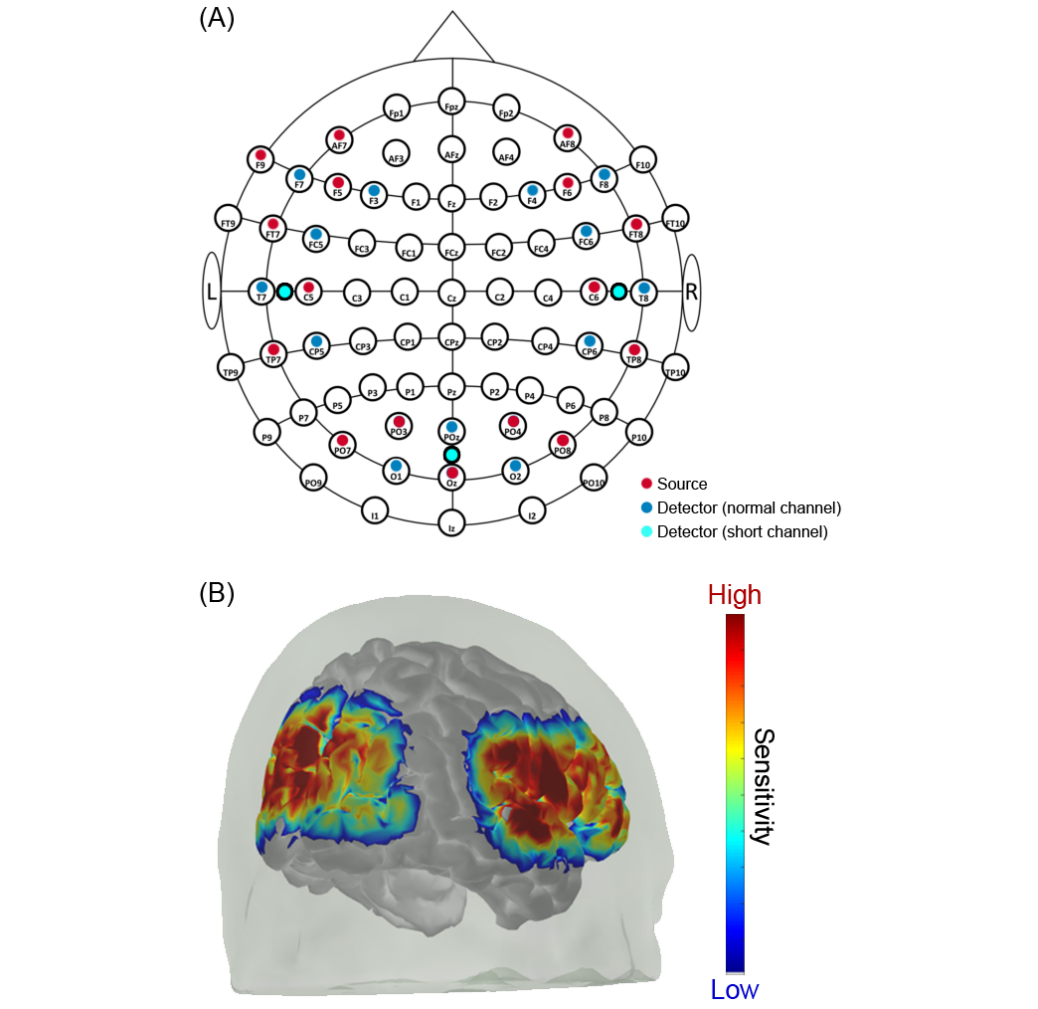
Functional near-infrared spectroscopy (fNIRS) montage. (A) Optode arrangement on the head. Sixteen sources (red circles) and 16 detectors (blue and cyan circles) will be placed on the scalp, forming a total of 43 channels. Three of the detectors (cyan circles) will be forming short-separation channels. (B) Sensitivity map of the optode arrangement.

The montage will consist of 16 sources and 16 detectors placed on the surface of the skull according to the international 10-10 system of electrode placement (Figure 2A) [54]. The source-detector pairs will result in a total of 43 channels in a multidistance setup: 3 of them are short-separation channels with a 15-mm interoptode distance, 4 are extra-long channels with a distance of 36-37 mm, and 36 are normal length channels that are approximately 30 mm apart. In a multidistance approach, shorter channels (15 mm) provide information about the interfering systemic signals in the outer cortex and longer channels (36+ mm) about brain activation in deep regions [55,56]. Practically, however, the signal-to-noise ratio may be poor in long distances, so in many cases we will not be able to use those channels. The Monte Carlo sensitivity simulation of all source-detector pairs is shown in Figure 2B and indicates a uniform sensitivity profile across the temporal, visual, and prefrontal cortical regions [57]. The sampling rate will be set to 14 Hz.

The optode holder cap will be assembled using the manufacturer's components (Holder kit, Shimadzu) and custom 3D printed parts (colored optode markers and stabilizers for different head sizes). The parts will be designed in a solid modelling software (SolidWorks 2019, Dassault Systemes) and printed using a 3D printer (Prusa i3 MK3S+, Prusa Research).

At the end of the experiment, we will digitize the position of all optodes with a depth sensing camera (Structure Sensor Pro, Occipital Inc) connected to an iPad (iPad Pro 2020, Apple Inc). The depth sensing camera will be set up for optimized scanning of dark objects with low ambient infrared light. The infrared exposure time, gain, and depth resolution will be set to the highest available settings so that the colored optode markers can be easily identified on the 3D scan.

##### Functional Near-Infrared Spectroscopy

**Figure 3:**
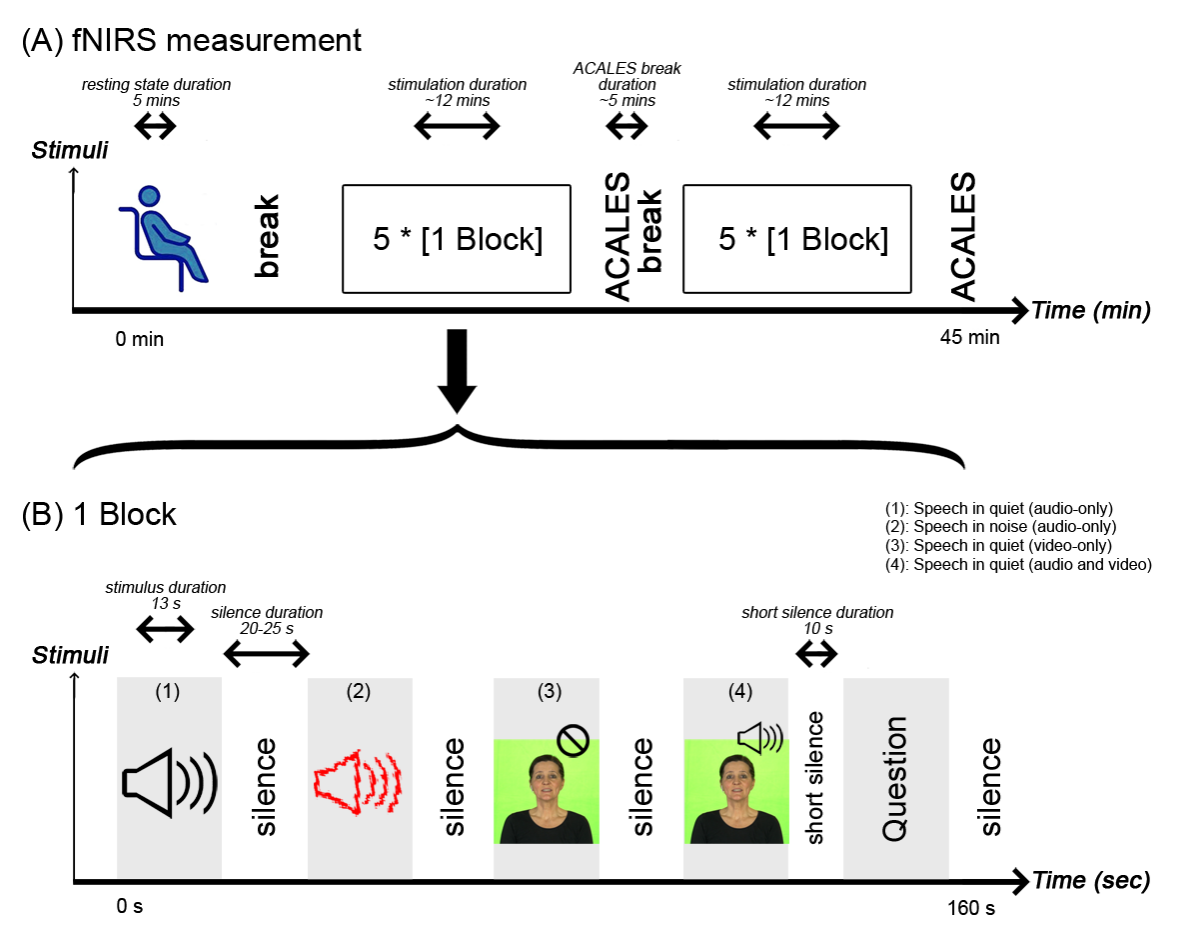
Functional near-infrared spectroscopy (fNIRS) measurement overview. (A) Following the resting state measurement, 2 x 5 counterbalanced blocks will be presented, with breaks in between. (A) A single block consists of a (1) speech in quiet (audio only), (2) speech in noise (audio only), (3) speech in quiet (video only), (4) speech in quiet (audio and video) stimulation, and an additional question.

During fNIRS recordings, we will instruct the participants to concentrate on the screen, follow the instructions, and reduce head movements. If the participants feel uncomfortable, an emergency button in front of them will be made available to stop the experiment. We will give all instructions both verbally and in writing. Before the recordings, the participants will conduct a short familiarization session with 4 example stimulations. Once the participant confirms that the task is understood, we will start the definitive recording. The functional recordings will begin with a 5-minute resting state period (Figure 3A). We will instruct the participant to sit still, close their eyes, and relax but try not to fall asleep. Then 2 stimulation sessions approximately 12 minutes each will follow. Between the 3 sessions (ie, the resting and the 2 stimulation sessions), the participants can take a break of their chosen duration.

##### Stimuli

As stimulus material, we will use a video version of the female OLSA test [40,58]. A single stimulus will consist of one sentence (eg, “Nina gives twelve red flowers”) which will be repeated 3 times. The duration of one stimulus will be 13 seconds, comparable to hemodynamic responses [59].

A single stimulation block will contain 4 different stimuli, presented in one of the following modalities in a counterbalanced order (Figure 3B): (1) speech in quiet (audio only), (2) speech in noise (audio only), (3) speech in quiet (video only, ie, lipreading), and (4) speech in quiet (audio and video). The stimulation will be followed by 20-25 seconds of nonstimulus interval, during which a white fixation point will be presented on a black screen. During the audio-only conditions, the same black screen will be displayed so that the participant will have no indication other than hearing whether the stimulation has already started or not.

At random points, participants will be asked to answer questions to ensure attention and monitor speech comprehension during the test. The questions will be displayed in the nonstimulus epoch, for which the nonstimulus interval will be shortened to 10 seconds. The questions will ask to repeat the correct name or number of the last sentence from 4 possible answers. For example, if the sentence is “Nina gives 12 red flowers,” the question is either ”How many red flowers?“ or ”Who gives 12 red flowers?“ To answer the question, the participant will have to select 1 of 4 choices: 2 randomly selected numbers/names from the OLSA sentence matrix (wrong answers), an option if the respondent is not sure of the answer (skipped answer), and the correct answer. For the previous question, a possible combination could be (1) ”Britta,” (2) “Nina,” (3) “Peter,” and (4) “I cannot decide.” The participant will select an option with the roller on the computer mouse and confirm the answer with a double click. In the previous example, the participant must select the second option (”Nina“). The questions and the answers will be randomly generated, and the position of the question within the blocks will also be randomly chosen.

The shortened nonstimulus interval of 10 seconds prior to the question window will allow us to evaluate the fNIRS responses. Therefore, the interleaved questions will not harm the overall effectiveness of the measurement. After the question is answered, the regular 20-25 second relaxation time will be applied to ensure that the brain responses return to baseline. Overall, 2 questions per modality will be asked, resulting in 8 questions throughout the entire fNIRS measurement.

Following the breaks, before the first stimulation, there will be a stimulus-free interval of 20 seconds. This will ensure homogeneity of responses, meaning that all stimuli are perceived under similar circumstances. Overall, 10 blocks will be presented, resulting in 10 responses per stimulation modality, and the total fNIRS measurement time will be around 45 minutes. At the beginning of every event (start/stop of a block, resting state, stimulation, question, answer), a trigger will be sent from the control computer to the fNIRS machine through the trigger-box and stored as an extra channel in the fNIRS raw data.

##### Listening Effort

Following 5 stimulation blocks, we will ask every participant to rate their listening effort to the different stimuli, their rating of fatigue and their level of mind-wandering (Figure 3A) [60-64]. To evaluate the listening effort, we will use Adaptive Categorical Listening Effort Scaling (ACALES) [65].

##### Data Management

All written source documents will be completed in a neat, legible manner to ensure accurate interpretation of data. For each participant, a case report form (CRF) will be maintained, including the participant number. In CRFs and other project-specific documents, participants are only identified by a unique participant number. fNIRS measurements will be stored in a closed research environment (REDCap, Vanderbilt University, Nashville, United States). The secure web application is running on a local server maintained and backed up by the University of Bern. All documents related to the study, including the CRFs will be considered as source data, and these will be stored at the measurement site in accordance with relevant standards.

#### Data Analysis

##### fNIRS Preprocessing

Data preprocessing will be performed in MATLAB (MathWorks) using the Homer2 (v2.3) [66] and NIRS [67] toolboxes. The signal quality will be checked based on the heart rate content of the signal, using a sliding window approach [68-71]. Channels and time points with insufficient signal quality will be removed. Short channel correction will be applied to the absorbance data, using short separation regression [56,72]. The motion artifacts will be removed with a WaveletFilter module of the NIRS toolbox [67]. The signal will be bandpass filtered between 0.01 and 0.12 Hz with the BandpassFilter function from the Homer toolbox [66]. Then, the absorbance data will be converted to concentration changes of oxygenated hemoglobin (HbO) and deoxygenated hemoglobin (HbR) in mMolar*cm using the following equations, as specified by the manufacturer based on the modified Beer-Lambert law [73]:

ΔHbO = (−1.4887) * Abs[780nm] + 0.5970 * Abs[805nm] + 1.4878 * Abs[830nm]

ΔHbR = 1.8545 * Abs[780nm] - 0.2394 * Abs[805nm] − 1.0947 * Abs[830nm]

A further correction step will be performed to reduce noise based on the principle that the concentration changes of oxygenated and deoxygenated hemoglobin should be negatively correlated [74].

##### Optode Positions

We will perform the postprocessing of the scans with a 3D mesh processing tool (MeshLab) and custom-written scripts (MathWorks) [75].

We will manually select the coordinates of the optodes and anatomical landmarks with MeshLab on the obtained 3D scans. The list of coordinates will then be exported and projected into Montreal Neurological Institute (MNI) space. The MNI coordinates will be displayed on the preoperative MRI scan of every CI-user participant, and the exact source of measured hemodynamic activation will be determined. Additionally, the mean and the standard deviation of optode coordinates will be calculated and reported as quality measure for optode fittings [55,76].

##### fNIRS Recordings

Data analysis will be performed in Python using the MNE-Toolbox [77] and MNE-NIRS package [78]. Individual epochs will be subtracted from the channel data, from t=0 seconds to *t*=24 seconds relative to the stimulus onset. The epochs will be baseline-corrected by subtracting the mean of the signal between t =−5 seconds and t=0 seconds. Using the Glover canonical hemodynamic response function [79] a design matrix for the general linear model (GLM) will be constructed [80,81]. After GLM fitting, the regression results will be stored. Following this, temporal and spatial features will be extracted from each epoch (amplitude, area under curve, peak latency, laterality, power). The regression results and the extracted features will be weight-averaged over ROIs by taking the inverse of the standard error of the GLM fit for each channel [67]. The data will be averaged over the participants, and group-level statistics will be calculated using correlation analysis and linear mixed-effects models.

##### Behavioral Data

The answers from the questionnaires will be digitized, and correlation analysis will be performed to reveal relations between the measured brain activation patterns and the evaluated questionnaires. Additionally, further behavioral data will be obtained from the triggers, such as reaction time to questions across the measurement as a measure of fatigue or response accuracy for each stimulation type as a measure of speech understanding.

## Results

The enrollment for the study described in this protocol started in August 2021. The first results are expected at the end of 2022.

## Discussion

The postoperative adaptive or maladaptive effect of existing cross-modal reorganization in CI candidates is a complex question. The available studies show contradictory findings. A recent review states that it is important to discuss the methodological aspects of such functional neuroimaging examinations [22-26,46,47,50]. One problem with measuring functional brain activation is that many variables must be considered. To better control these variables, we present hereby an audiovisual speech comprehension task that fulfills the 6 points outlined below.

First, the test should be deducible from clinically established hearing tests. We used the video version of a widely used clinical test (Oldenburg Sentence Test) [58]. Functional brain activation patterns can therefore be correlated with clinical findings. These results are easier to interpret than custom-made speech materials [23,46,47,50]. Our employed stimulation design consists of complete sentences, which reflect everyday life and real language comprehension much better than nonspeech auditory stimuli or speech snippets [13,23,25,28,46,47]. Before conducting the fNIRS experiment, we will repeat clinical speech comprehension tests (ie, Freiburg monosyllabic test, Oldenburg Sentence Test). This enables a clear grouping of the CI participants into good and poor performers. During the fNIRS experiment, we will continue to assess speech comprehension in 4 different situations (ie, speech in quiet, speech in noise, visual-speech, audiovisual speech) with interleaved comprehension questions. This allows us to maintain attention and monitor speech comprehension while measuring brain activity. This advantage has only been applied by one research group [22,24]. To assess listening effort during the fNIRS task, we will use a validated questionnaire (ie, ACALES) [65]. Listening effort in CI users is an active topic of discussion [82]; its possible influence on the measured cortical activation, to the best of our knowledge, has never been reported before. To describe the subjective hearing perception in their daily lives, participants will complete validated questionnaires (ie, SSQ-12) on the day of the test [38]. We will conduct our tests in a validated audio chamber (as used in clinically performed hearing tests).

Second, the task should induce maximal cortical activation (and thus allow reproducible recognition of activation patterns). We use an optimized counterbalanced block design. The duration of 1 stimulus will be 13 seconds, and the interstimulus break will be between 20 and 25 seconds, comparable to hemodynamic responses [49,59]. Our task requires the active participation of the participants. Previous studies have shown that this can significantly increase brain activation [63,64]. Furthermore, we mitigate mind wandering and fatigue by filling out validated questionnaires [60-62]. As far as we know, in persons with hearing impairments, this has never been reported in the context of fNIRS measurements. To avoid fatigue (which can lead to decreased brain activation), we keep the fNIRS task as short as possible. Additionally, participants can take 2 breaks of self-selected duration.

Third, it should be in alignment with the international 10-10 system of electrode placement, using optimally spaced optode positions with maximal coverage over the responsible brain regions. Short separation channels should allow noise reduction. Our optode placement covers the following brain regions: superior temporal gyrus (STG), primary visual cortex (V1), visual association cortex (V2), left inferior frontal gyrus (LIFG), middle temporal gyrus (MTG), and middle frontal gyrus (MFG). This allows us to study not only audiovisual activations but also speech perception in noise, the effects of fatigue, and activity related to higher-order cortical processing. Many other studies have not had the opportunity to cover such a wide range of cortical regions, mostly due to hardware limitations [22-25,50]. We use the Edinburgh Handedness Inventory to control for handedness, which might affect the laterality of brain activation [33-35]. We also perform a spatial registration of optode positions to increase reproducibility. Furthermore, these measured positions can be projected into MNI space and displayed on MRI images. In the diagnostic workup, MRI are routinely performed prior to CI surgery. The method will allow a more accurate localization of hemodynamic responses compared to atlas-based approaches [55,76].

Additionally, we use a multidistance channel setup. The optodes of the regular channels are ~30 mm apart from each other. Additional short channels with a 15-mm interoptode distance over the auditory and visual cortex provide extracerebral information to remove confounding systemic signals. It is recommended to use a systemic physiology controlled fNIRS approach, although this has rarely been applied in previous studies [55].

Fourth, it should be time efficient to avoid fatigue due to experiment duration. The longest task the participants will be required to complete will last 12 minutes, and the total measurement time will be around 45 minutes. Regular breaks will be provided, and the total duration of the experiment is expected to be around 120-150 minutes.

Fifth, it should be suitable for participants with normal hearing, hearing impairments, and those using CIs. The audio material is presented through a loudspeaker, so the task is suitable for people with normal hearing as well as for hearing aid and CI users. Alternatively, an insert earphone or a direct CI audio input simulation would be feasible. However, these 2 approaches have the disadvantage that the 3 aforementioned groups cannot not be stimulated identically. Our optode placement was chosen to allow for easy attachment of the implant coil.

Sixth, it should be reproducible by other research groups. The audiovisual version of the OLSA was published in 2021 and is now accessible [58]. Moreover, we are happy to share our setup upon request.

In summary, the proposed audiovisual speech comprehension task will help us understand neural correlates to speech understanding. In the first stage, we will perform these measurements postoperatively to better understand the corresponding neuronal networks with an activated implant. In the subsequent stage, we will perform the measurements pre- and postoperatively to make prognostic calculations. The comprehensive study will have the potential to provide additional prognostic information beyond the conventional clinical standards regarding the underlying plastic brain changes of a person with hearing impairment. Our study will facilitate more precise indication criteria for cochlear implantation and a better planning of rehabilitation.

## Appendix

Multimedia Appendix 1 Neurological, cardiac, psychiatric, or other major diseases used as exclusion criteria.

## Abbreviations

ACALES: Adaptive Categorical Listening Effort Scaling
CI: cochlear implant
CRF: case report form
EEG: electroencephalography
fNIRS: functional near-infrared spectroscopy
GLM: general linear model
HbO: oxygenated hemoglobin
HbR: deoxygenated hemoglobin
HL: hearing level
LIFG: left inferior frontal gyrus
MFG: and middle frontal gyrus
MNI: Montreal Neurological Institute
MRI: magnetic resonance imaging
MTG: middle temporal gyrus
OLSA: Oldenburg Sentence Test
PTA: pure-tone average
ROI: region of interest
SPL: sound pressure level
SSQ-12: Speech, Spatial, and Qualities
STG: superior temporal gyrus
TTL: Transistor-Transistor Logic
V1: primary visual cortex
V2: visual association cortex
