# Correction: Neural Activity During Audiovisual Speech Processing: Protocol For a Functional Neuroimaging Study

**DOI:** 10.2196/40527

**Published:** 2022-06-28

**Authors:** András Bálint, Wilhelm Wimmer, Marco Caversaccio, Stefan Weder

**Affiliations:** 1 Department of Otorhinolaryngology, Head and Neck Surgery Inselspital, Bern University Hospital, University of Bern Bern Switzerland; 2 Hearing Research Laboratory ARTORG Center for Biomedical Engineering Research, University of Bern Bern Switzerland

In “Neural Activity During Audiovisual Speech Processing: Protocol for a Functional Neuroimaging Study” (JMIR Res Protoc 2022;11(6):e38407), the authors noted one error.

The originally published article appeared with an incorrect Abstract. In the corrected version, the Abstract is updated as follows:

**Background:** Functional near-infrared spectroscopy (fNIRS) studies have demonstrated associations between hearing outcomes after cochlear implantation and plastic brain changes. However, inconsistent results make it difficult to draw conclusions. A major problem is that many variables need to be controlled. To gain further understanding, a careful preparation and planning of such a functional neuroimaging task is key.

**Objective:** Using fNIRS, our main objective is to develop a well-controlled audiovisual speech comprehension task to study brain activation in individuals with normal hearing and hearing impairment (including cochlear implant users). The task should be deductible from clinically established tests, induce maximal cortical activation, use optimal coverage of relevant brain regions, and be reproducible by other research groups.

**Methods:** The protocol will consist of a 5-minute resting state and 2 stimulation periods that are 12 minutes each. During the stimulation periods, 13-second video recordings of the clinically established Oldenburg Sentence Test (OLSA) will be presented. Stimuli will be presented in 4 different modalities: (1) speech in quiet, (2) speech in noise, (3) visual only (ie, lipreading), and (4) audiovisual speech. Each stimulus type will be repeated 10 times in a counterbalanced block design. Interactive question windows will monitor speech comprehension during the task. After the measurement, we will perform a 3D scan to digitize optode positions and verify the covered anatomical locations.

**Results:** This paper reports the study protocol. Enrollment for the study started in August 2021. We expect to publish our first results by the end of 2022.

**Conclusions:** The proposed audiovisual speech comprehension task will help elucidate neural correlates to speech understanding. The comprehensive study will have the potential to provide additional information beyond the conventional clinical standards about the underlying plastic brain changes of a hearing-impaired person. It will facilitate more precise indication criteria for cochlear implantation and better planning of rehabilitation.

The correction will appear in the online version of the paper on the JMIR Publications website on June 28, 2022, together with the publication of this correction notice. Because this was made after submission to PubMed, PubMed Central, and other full-text repositories, the corrected article has also been resubmitted to those repositories.

