# Supplementary material for: Neural Activity During Audiovisual Speech Processing: Protocol For a Functional Neuroimaging Study
Source: JMIR Res Protoc. 2022 Jun 21;11(6):e38407. doi: 10.2196/38407 (PMC9239541; doi:10.2196/38407)
Supplement: Multimedia Appendix 1 [file resprot_v11i6e38407_app1.pdf]

Supplementary table S1. Neurological, cardiac, psychiatric, or other major diseases used as exclusion criteria.

**Medical History**
Traumatic brain injury
Epilepsy
Tumor
Parkinson’s disease
Dementia / Alzheimer
Stroke
Schizophrenia
Multiple Sclerosis
Heart disease
Aneurysms
Tremor
Seizure
Diabetes mellitus
